# Side impact motor vehicle crashes: driver, passenger, vehicle and crash characteristics for fatally and nonfatally-injured rear-seated adults

**DOI:** 10.1186/s40621-016-0088-1

**Published:** 2016-10-03

**Authors:** Chang Liu, Joyce C. Pressley

**Affiliations:** 1 Columbia University Mailman School of Public Health Department of Epidemiology, 722 West 168th St., Suite 812G, New York, NY 10032 USA; 2 Columbia University Mailman School of Public Health Department of Health Policy and Management, 722 West 168th St., Suite 812G, New York, NY 10032 USA; 3 Center for Injury Epidemiology and Prevention at Columbia University Mailman School of Public Health, 722 West 168th St., Suite 812G, New York, NY 10032 USA

**Keywords:** Adults, Rear-seated occupant safety, Motor vehicle crash and seatbelts

## Abstract

**Background:**

Most studies of rear-seated occupants have focused on or included pediatric occupants which may not translate to adults. This study examines passenger, driver, vehicle and crash characteristics for rear-seated adult occupants involved in side crashes.

**Methods:**

The National Automotive Sampling System General Estimates System (NASS/GES) for calendar years 2011–2014 was used with accompanying weights to examine the occupant, vehicle and crash characteristics associated with injury in rear-seated adults (*n* = 395,504) involved in a side crash. A weighted subpopulation analysis includes occupants travelling in a vehicle with an IIHS safety rating (*n* = 39,208), which was used to control for vehicle safety. Statistical analysis used Chi-square tests and multilevel multivariable logistic regression. Unadjusted and adjusted odds ratios (ORs) are reported with 95 % confidence intervals (95 % CIs).

**Results:**

Rear-seated occupants on the same side as the crash impact were more likely to be severely/fatally injured than occupants seated on the opposite side (Multivariable adjusted OR: 2.54, 95 % CI: 2.31–2.79), as were those in angle crashes (Multivariable adjusted OR: 10.85, 95 % CI: 9.24–12.73). Rear-seated occupants of belted drivers were 3.28 times more likely to be belted compared to rear-seated occupants of an unbelted driver. In a subpopulation analysis of all same-side crashes, unrestrained occupants were 5.96 times more likely to be severely/fatally injured compared to restrained occupants.

**Conclusion:**

Restraint use was protective for rear-seated adult occupants involved in side crashes, including those in same-side crashes. Angle and same-side crashes are associated with increased injury severity.

## Background

Rear-seated adult occupants involved in a fatal crash who are belted have lower mortality than unbelted rear-seated occupants except possibly those in same-side crashes (Raneses and Pressley 2015). This is consistent with reports of frontal same-side crashes where it has been noted that the most severe injury comes from contact with the adjacent side structure, frontal components and ejection (Farmer et al. 1997; Sunnevang et al. 2009). Being belted is protective for ejection, but may not protect against occupant compartment infringement.

Same side crashes are reported to have higher serious injury and fatality rates compared to other crash types (Samaha and Elliott 2003). Front-seated drivers of vehicles with a safety rating of “good” are reported to be 70 % less likely to die when involved in side crashes compared to vehicles in the poorly rated category (Teoh and Lund 2011), but this was not seen in a small sample of older rear-seated adult occupants involved in fatal collisions (Raneses and Pressley 2015).

Rear-seated occupants may be more vulnerable to injury than front-seated occupants who have more advanced safety technology, suggesting that more attention is needed for rear-seated occupants (Durbin et al. 2015). Most studies of rear-seated occupants have focused on or included pediatric populations restrained in child restraints or booster seats, which may not be generalizable to adult rear-seat safety (Evans and Frick 1988; Smith and Cummings 2006; Mayrose and Priya 2008; Stewart et al. 2013; Zhu et al. 2007). The aims of this study are to examine injury outcomes for rear-seated adult occupants involved in a side crash with respect to injury severity and: 1) driver, passenger, vehicle and crash characteristics; 2) seating position and point of impact; and 3) whether and to what extent injury severity is mitigated by other characteristics, such as vehicle side crash safety ratings and striking vehicle characteristics. Further elucidation of associated factors may identify areas of intervention to further reduce motor vehicle occupant injury and mortality.

## Methods

### Data source

Data were obtained from the National Automotive Sampling System General Estimates System (NASS/GES) for calendar years 2011–2014 (NHTSA 2015). NASS/GES is a nationally representative sample of law enforcement reported motor vehicle crashes. The Police Accident Reports (PAR) comprising NASS/GES are chosen from 60 areas that reflect the geography, roadway mileage, population and traffic density of the U.S. general population using multi-stage sampling methods. The first stage is a sample of geographic areas, called Primary Sampling Units (PSUs) from the United States, with the second stage of the design being a sample of jurisdictions within each PSU. The final stage uses a selection of crashes within the sampled jurisdiction. A national weight is included in a data file for each PAR and is called “WEIGHT”, which is the product of an inverse of the probabilities of selection at each of the three stages. NASS/GES contains person-, vehicle-, and crash-level variables, including drivers’ and passengers’ age and gender, seating position, restraint use, severity of injury, vehicle body type, initial impact point, type of crash and other related variables for all types of crashes. The publicly available Insurance Institute for Highway Safety (IIHS 2014a) crash ratings were added into NASS/GES using the common variables of vehicle make, model and model year.

### Study population

**Fig. 1:**
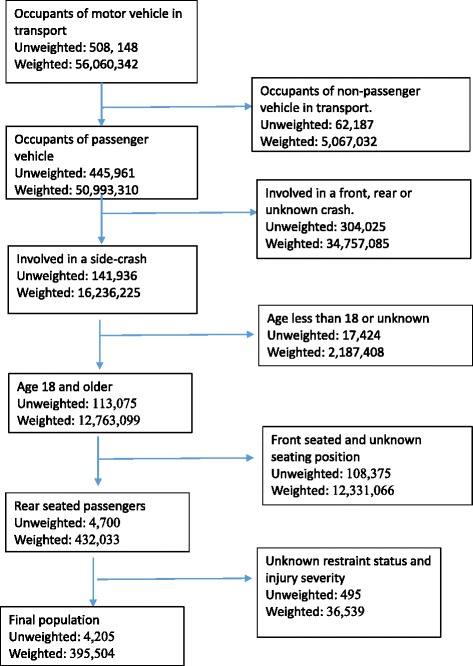
Population flow diagram for NASS/GES, 2011–2014

The study population included 4,205 (weighted: 395,504) rear-seated occupants aged 18 years and older who were travelling in a four-wheeled passenger vehicle involved in a side crash between 2011 and 2014 (Fig. 1). The subpopulation analysis of vehicles that had an IIHS safety rating included 411 (weighted 39,208) occupants involved in a same-side crash. The subpopulation was used to control for vehicle characteristics while investigating belt status, seating position, point of impact and injury severity.

### Variable classification

#### Occupant characteristics

**Demographics.** Passenger age was examined both as a continuous and categorical variable. Age was categorized as 18–24, 25–44, 45–64 and older than 65 years in the descriptive analysis and was modeled as a continuous variable with a quadratic term (Table 2). Driver age was categorized as 15–19, 20–44, 45–64 and older than 65. Gender was categorized as male or female as provided in NASS/GES. Race was not available in the NASS/GES dataset.

**Restraint use.** Restraint use was dichotomized as: 1) not belted; and 2) belted (shoulder belt, lap belt, shoulder and/or lap belt).

**Driver alcohol involved.** The driver was considered to have alcohol present if police reported alcohol or the driver had a blood alcohol concentration (BAC) of 0.01 or higher.

**Injury severity.** Injury severity was examined as a dichotomous variable: 1) no or minor/possible injury; or 2) serious/fatal injury. Injury severity was reported by the attending law enforcement officer using KABCO coding scheme. Fatality was ascertained up to 30 days post crash from other sources. The KABCO scale, developed by the National Safety Council (NSC), is frequently used by law enforcement for classifying injuries: K – Fatal; A – Incapacitating injury; B – Nonincapacitating injury; C – Possible injury; and O – No injury (National Safety Council 1990).

#### Vehicle characteristics

**Vehicle model year.** Vehicle model year was examined both as a continuous variable from 1970–2014 and categorized to represent the eras of vehicle improvements: 1970–1993, 1994–1997, 1998–2004, 2005–2008, and 2009 and later (Ryb et al. 2011).

**Model type.** Four-wheeled passenger vehicle type was categorized as: car, SUV, van, or pick-up truck based on the ‘Body Type’ variable provided in NASS/GES. Rear-seated occupants being transported in buses, large trucks, ATVs, farm equipment, motor homes and large limousines were excluded.

**Vehicle safety rating.** The vehicle side safety rating was assessed using publicly available IIHS safety ratings. In IIHS side impact rating, nine aspects of performance are separately rated as good, acceptable, marginal or poor and then combined to produce an overall side impact rating (IIHS 2014b). Vehicle crash test ratings, categorized as good, acceptable, marginal, poor or unrated, were merged into NASS/GES data by linking variables on make, model and model year of the vehicle which were common in both data sets.

#### Crash characteristics

**Fig. 2:**
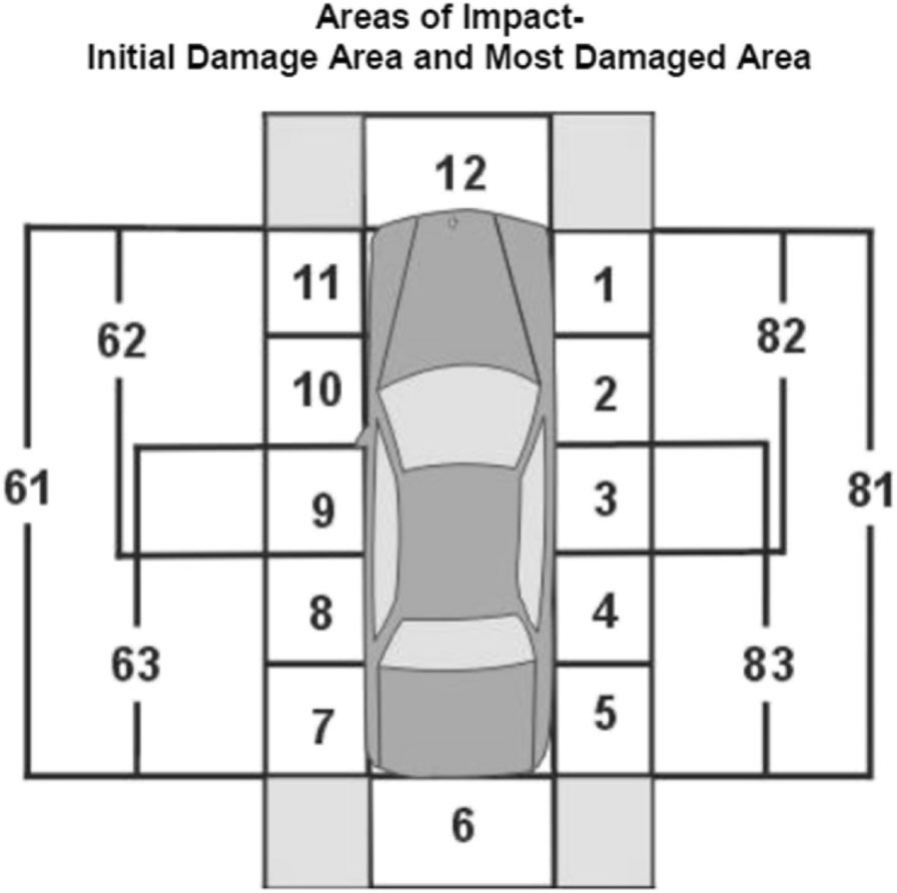
Initial point of impact for rear seated adult occupants, NASS/GES, 2011–2014

**Side crash.** Side crash is defined based on the initial point of impact, which was categorized based on clock points as shown in Fig. 2 (NHTSA 2015). We examined side impact crash points (1–5, 7–11, 81, 82, 83, 61, 62, 63) as side crashes.

**Same side crash.** Same side crash is defined as having the initial point of impact and the passenger seating position on the same side of the impacted vehicle.

**Manner of collision.** Manner of collision is categorized in NASS/GES based on first harmful event and grouped into 5 categories: 1) not a collision with vehicle in transport; 2) angle; 3) sideswipe-same direction; 4) sideswipe-opposite direction and 5) other.

**Ejection.** Ejection is categorized as: ejected (fully or partially) and not ejected.

**Rollover.** Rollover is categorized as dichotomous according to whether or not a rollover or overturn was associated with this vehicle.

**Excessive speed.** Excessive speed was analyzed using a dichotomous variable. A vehicle was categorized as speeding if the driver was charged with a speeding violation or if the travel speed of the vehicle was reported to be above the posted speed limit. Due to large quantities of missing data, actual miles per hour traveled were not available for analysis.

**Weekday/weekend.** A social weekend was defined as 6:00 p.m. Friday to 6:00 p.m. Sunday. Weekend versus weekday was analyzed as a dichotomous variable.

**Day/Night.** A dichotomous variable was used to distinguish daytime (6:00 a.m. to 5:59 p.m.) and nighttime (6:00 p.m. to 5:59 a.m.).

### Statistical analysis

Bivariable descriptive analyses used the Chi-square test to examine passenger and driver age categories, gender, restraint use, vehicle type and manner of collision and other key variables for rear-seated occupants. SAS SURVEYLOGISTICS was used for analysis of weighted data in a subpopulation analysis for passengers involved in same-side crashes. The associations of injury severity, occupant, crash and vehicle characteristics were analyzed using multilevel logistic regression to generate odds ratios with 95 % confidence intervals. Since there could be multiple passengers in the same vehicle, the SAS GLIMMIX procedure (SAS Institute Inc 2014) with random effects was used to generate odds ratios with 95 % confidence intervals using multilevel models that controlled for violation of the assumption of independence associated with multiple rear-seated occupants in the same vehicle. A derived group variable that created a unique vehicle-specific number was used to control for clustering of passengers in the same vehicle. Analyses were performed on variables weighted by primary sampling units, strata and assigned weight for each PAR. Final models were age and gender-adjusted with model selection based on variables whose unadjusted odds ratios were significant at *p* ≤ 0.2. All analyses were conducted in SAS 9.4.

## Results

The study population consisted of 4,205 (weighted: 395,504) adult rear-seated occupants with a mean of 1.3 passengers aged 18 years or older per vehicle. The majority (77.8 %) of vehicles had 1 adult passenger, 17.5 % had 2 adult passengers and 4.6 % had 3 or more adult passengers seated in the rear.

### Occupant characteristics

#### Passenger age and gender

The majority of occupants involved in a crash were aged between 18–44 years (Table 1). In analyses using age as a continuous variable, older occupants were more likely to have a serious/fatal injury compared to younger passengers. Female occupants were more likely to be seriously/fatally injured compared to males in unadjusted analyses but gender differences were reduced after adjusting for independent predictors of injury severity (OR: 1.07, 95 % CI: 0.98–1.18) (Table 2).

**Table 1:** Passenger, driver, vehicle and crash characteristics for rear-seated passengers involved in side crashes, NASS/GES 2011–2014

	Belted		Unbelted		Total	
	No/Minor injury	Severe/Fatal Injury	No/Minor injury	Severe/Fatal Injury		
	n (%)	n (%)	n (%)	n (%)	n (%)	Chisq (*p*-value)^a^
Total^b, c^	361,170 (91.3)	3,924 (1.0)	27,918 (7.1)	2,492 (0.6)	395,504 (100.0)	
Passenger Characteristics (*n*=395,504)						
Age						0.2 (0.95)
18–24	154,228 (42.7)	1,659 (42.3)	15,302 (54.8)	909 (36.5)	172,098 (43.5)	
25–44	111,107 (30.8)	1,043 (26.6)	8,833 (31.6)	1,083 (43.5)	122,067 (30.8)	
45–64	62,810 (17.4)	745 (19.0)	2,980 (10.7)	426 (17.1)	66,960 (16.9)	
65+	33,026 (9.1)	477 (12.2)	803 (2.9)	74 (3.0)	34380 (8.7)	
Gender						0.006 (0.92)
Male	164,163 (45.6)	1,623 (41.5)	13,047 (46.7)	1,234 (49.5)	180,067 (45.6)	
Seating Position						0.07 (0.92)
Left seated	143,658 (39.9)	1,556 (39.9)	11,069 (40.7)	914 (38.8)	157,197 (39.9)	
Middle seated	25,099 (7.0)	118 (3.0)	3,515 (12.9)	330 (14.0)	29,062 (7.4)	
Right seated	191,408 (53.1)	2,228 (57.1)	12,595 (46.3)	1,111 (47.2)	207,341 (52.7)	
Driver Characteristics (*n*=284,554)						
Age						0.9 (0.48)
15–19	32,639 (13.6)	337 (12.0)	2,791 (15.7)	225 (11.8)	38,041 (13.4)	
20–44	129,185 (53.8)	1,339 (48.0)	9,699 (54.7)	1,274 (67.1)	153,703 (54.5)	
45–64	57,849 (24.1)	715 (25.6)	4,486 (25.3)	349 (18.4)	68,002 (24.1)	
65+	20,287 (8.5)	396 (14.1)	731 (4.1)	51 (2.6)	22,287 (7.9)	
Gender						1.5 (0.17)
Male	143,479 (59.8)	1,377 (49.1)	12,189 (68.1)	999 (52.5)	170,237 (59.8)	
Restraint Status						27.6 (<0.0001)
Yes	237,425 (98.3)	2,709 (97.2)	14,334 (79.3)	1,321 (73.6)	20,998 (97.7)	
Alcohol involved						26.8 (<0.0001)
Yes	3,461 (1.5)	115 (4.5)	706 (4.3)	393 (23.1)	5,795 (2.2)	
Vehicle Characteristics						
Vehicle model year						5.9 (0.025)
<1994	9,258 (2.6)	60 (1.5)	555 (2.0)	64 (2.6)	9,937 (2.5)	
1994–1997	21,177 (5.9)	166 (4.2)	1,754 (6.3)	110 (4.4)	23,207 (5.9)	
1998–2004	138,809 (38.6)	2,245 (57.2)	14,872 (53.3)	1,245 (50.0)	157,171 (39.9)	
2005–2008	93,075 (25.9)	856 (21.8)	5,122 (18.3)	483 (19.4)	99,536 (25.3)	
2009–2014	97,506 (27.1)	596 (15.2)	5,616 (20.1)	590 (23.7)	104,308 (26.5)	
Vehicle type						4.8 (0.073)
Car	207,252 (57.4)	2,690 (68.5)	17,008 (60.9)	1,614 (64.8)	228,564 (57.8)	
SUV	71,244 (19.7)	855 (21.8)	5,478 (19.6)	439 (17.6)	78,016 (19.7)	
Van	37,633 (10.4)	240 (6.1)	3,207 (11.5)	149 (6.0)	41,228 (10.4)	
Pickup	45,041 (12.5)	140 (3.6)	2,225 (8.0)	290 (11.6)	47,686 (12.1)	
Crash Rating						
Side crash rating						8.9 (0.018)
Unrated	288,448 (79.9)	3,200 (81.6)	22,465 (80.5)	1,662 (66.7)	315,775 (79.8)	
Poor	20,289 (5.6)	230 (5.9)	2,530 (9.1)	89 (3.6)	23137 (5.9)	
Marginal	6,450 (1.8)	54 (1.4)	729 (2.6)	160 (6.4)	7,393 (1.9)	
Acceptable	6,717 (1.9)	210 (5.4)	411 (1.5)	209 (8.4)	7,548 (1.9)	
Good	39,266 (10.9)	230 (5.9)	1,784 (6.4)	372 (14.9)	41,651 (10.5)	
Crash Characteristics						
Time of crash						0.02 (0.88)
Day	216,322 (60.0)	2,592 (66.1)	12,681 (45.4)	1,291 (51.8)	232,886 (59.0)	
Night	144,285 (40.0)	1,332 (33.9)	15,237 (54.6)	1,201 (48.2)	162,054 (41.0)	
Week of Day						0.04 (0.78)
Weekday	228,083 (63.2)	2,443 (62.3)	17,709 (63.4)	1,526 (61.3)	249,761 (63.2)	
Weekend	133,087 (36.8)	1,481 (37.7)	10,209 (36.6)	966 (38.7)	145,743 (36.9)	
Crash Side						4.2 (0.010)
Same side	169,036 (46.9)	2,272 (58.2)	11,455 (42.1)	1,374 (58.4)	184,137(46.8)	
Middle seated	25,099 (7.0)	118 (3.0)	3,515 (12.9)	330 (14.0)	29,062 (7.4)	
Opposite side	166,030 (46.1)	1,512 (38.7)	12,209 (44.9)	650 (27.6)	180,402 (45.8)	
Crash Type						23.9 (<0.0001)
Single vehicle	44,837 (13.0)	726 (18.6)	5,063 (18.9)	1,017 (40.8)	51,643 (13.6)	
Angle	156,095 (45.2)	2,630 (67.4)	13,297 (49.5)	962 (38.6)	172,984 (45.6)	
Sideswipe-same direction	129,184 (37.4)	498 (12.8)	6,815 (25.4)	485 (19.4)	136,982 (36.1)	
Sideswipe-opposite direction	15,605 (4.5)	47 (1.2)	1,681 (6.3)	28 (1.1)	17,361 (4.6)	
Striking vehicle						17.7 (<0.0001)
No striking vehicle	37,264 (10.7)	579 (15.9)	4,674 (17.7)	998 (40.3)	43,515 (11.4)	
Multiple vehicle	21,133 (6.1)	252 (6.9)	708 (2.7)	247 (10.0)	22,341 (5.9)	
Smaller/similar	201,727 (57.8)	1,832 (50.3)	13,552 (51.3)	569 (23.0)	217,680 (57.1)	
Larger	88,706 (25.4)	982 (26.9)	7,494 (28.4)	661 (26.7)	97,843 (25.7)	
Rollover						52.6 (<0.0001)
Yes	6,315 (1.7)	369 (9.4)	1,994 (7.1)	640 (25.7)	9,318 (2.4)	
Ejection						269.7 (<0.0001)
Yes	52 (0.0)	84 (2.3)	405 (1.6)	596 (24.4)	1,137 (0.3)	
Speed related						27.1 (<0.0001)
Yes	15,780 (4.4)	345 (8.9)	1,358 (5.0)	759 (30.8)	18,242 (4.7)	

^a^Chi-square and p value expressed are for the relationship between the left-side variable and severe/fatal injury

^b^Percentage for Total are row percentages; all the other are column percentages

^C^Total in each stratum are different due to missing values

**Table 2:** Serious/fatal injury of rear-seated adult passengers involved in a side crash: Unadjusted and adjusted odds ratios (with 95 % CIs) using multilevel logistic regression, NASS/GES, 2011–2014

	Unadjusted		Belt status adjusted		Multivariable adjusted with rollover^a^		Multivariable adjusted without rollover^b^	
Passenger Characteristics								
Age, continuous	0.99	(0.97, 1.01)	0.99	(0.97, 1.01)	1.02	(1.02, 1.02)	1.02	(1.02, 1.02)
Gender								
Male		ref		ref		ref		ref
Female	5.38	(4.31, 6.72)	2.51	(2.16, 2.92)	1.43	(1.27, 1.60)	1.07	(0.98, 1.18)
Seating position								
Left		ref		ref				
Middle	0.25	(0.17, 0.35)	0.01	(0.01, 0.02)				
Right	1.28	(1.08, 1.51)	0.98	(0.84, 1.14)				
Restraint Status								
Yes		ref				ref		ref
No	9.44	(3.02, 29.50)		-	22.95	(18.36, 28.68)	10.55	(9.12, 12.21)
Driver Characteristics								
Age								
15–19		ref		ref				
20–44	0.80	(0.71, 0.90)	1.23	(0.88, 1.72)				
45–64	0.47	(0.41, 0.55)	1.29	(0.89, 1.86)				
65+	0.77	(0.65, 0.92)	1.40	(0.88, 2.23)				
Gender								
Male		ref		ref		ref		ref
Female	1.59	(1.29, 1.96)	1.77	(1.40, 2.22)	2.36	(2.08, 2.67)	2.25	(2.04, 2.49)
Restraint Status								
No		ref		ref		ref		ref
Yes	4.24	(3.42, 5.26)	2.36	(1.63, 3.41)	6.32	(4.75, 8.41)	2.35	(1.84, 3.00)
Alcohol involved								
No		ref		ref		ref		ref
Yes	44.95	(9.39, 215.19)	1.60	(1.30, 1.97)	13.76	(10.38, 18.25)	11.98	(9.57, 14.99)
Vehicle Characteristics								
Model year, continuous	0.95	(0.91, 0.99)	0.96	(0.93, 0.98)				
Model year, categorical								
<1994		ref		ref				
1994–1997	1.72	(0.85, 3.48)	1.05	(0.51, 2.17)				
1998–2004	2.18	(1.15, 4.14)	1.83	(0.97, 3.43)				
2005–2008	1.17	(0.61, 2.26)	1.06	(0.55, 2.02)				
2009–2014	1.02	(0.53, 1.96)	0.92	(0.48, 1.76)				
Vehicle type								
Car		ref		ref		ref		ref
SUV	0.80	(0.61, 1.06)	0.89	(0.73, 1.09)	0.40	(0.34, 0.47)	0.47	(0.41, 0.54)
Van	0.51	(0.34, 0.78)	0.48	(0.35, 0.67)	0.41	(0.33, 0.51)	0.48	(0.40, 0.56)
Pickup	0.43	(0.29, 0.66)	0.38	(0.27, 0.54)	0.29	(0.23, 0.37)	0.22	(0.18, 0.28)
Crash Characteristics								
Crash Side								
Same side	2.47	(2.08, 2.94)	2.78	(2.39, 3.23)	2.76	(2.48, 3.08)	2.54	(2.31, 2.79)
Middle seated	0.47	(0.33, 0.67)	0.01	(0.00, 0.01)	0.64	(0.51, 0.80)	0.74	(0.60, 0.90)
Opposite side		ref		ref		ref		ref
Crash Type								
Single vehicle	19.35	(15.69, 23.87)	13.63	(11.21, 16.58)	4.50	(3.59, 5.64)	5.23	(4.32, 6.32)
Angle	10.96	(9.28, 12.94)	8.71	(7.40, 10.26)	13.81	(11.15, 17.11)	10.85	(9.24, 12.73)
Sideswipe-same direction	0.18	(0.11, 0.27)	0.18	(0.13, 0.27)	0.23	(0.15, 0.36)	0.27	(0.18, 0.41)
Sideswipe-opposite direction		ref		ref		ref		ref
Striking vehicle								
No striking vehicle	4.26	(1.89, 9.60)	2.55	(2.01, 3.23)				
Multiple vehicle	0.90	(0.33, 2.46)	1.83	(1.27, 2.62)				
Smaller/similar		ref		ref				
Larger	1.64	(0.87, 3.09)	1.45	(1.17, 1.78)				
Rollover								
No		ref		ref		ref		
Yes	31.66	(7.85, 127.63)	23.17	(5.22, 102.85)	42.40	(31.25, 57.53)		
Speed related								
No		ref		ref		ref		ref
Yes	5.99	(2.06, 17.45)	2.14	(1.47, 3.11)	7.70	(6.00, 9.88)	5.70	(4.66, 6.97)
Time of crash								
Day		ref		ref				
Night	0.96	(0.78, 1.19)	0.86	(0.64, 1.01)				
Week of Day								
Weekday		ref		ref				
Weekend	1.09	(0.89, 1.33)	1.01	(0.86, 1.19)				

^a^Includes passengers that experienced a rollover

^b^Passengers (n=9,318) involved in a rollover were excluded in the model

#### Passenger seating position

In general, right-seated occupants were more likely to be seriously/fatally injured compared to left-seated occupants (OR: 1.28, 95 % CI: 1.08–1.51), while middle-seated occupants were less likely to be seriously/fatally injured (OR: 0.25, 95 % CI: 0.17–0.35) in the unadjusted model (Table 2). Middle-seated occupants were less likely to be belted (86.5 %) compared to left-seated (92.4 %) or right-seated (93.3 %) occupants. After controlling for restraint use, the right-seated occupants were no longer more likely to be seriously/fatally injured compared to the left-seated (OR: 0.98, 95 % CI: 0.84–1.14), but the middle-seated occupants were still less likely to be seriously/fatally injured (OR: 0.01, 95 % CI: 0.01–0.02) (Table 2).

#### Restraint use and mortality

The majority of rear-seated occupants were reported to be belted (92.3 %). Belted occupants were less likely to have a severe/fatal injury compared to those who were not belted. This was seen for same-side, middle-seated and opposite-side crashes. Restraint use was protective after controlling for crash type and seating position, with unbelted occupants more likely to have serious/fatal injury (OR: 10.55, 95 % CI: 9.12–12.21) (Table 2).

#### Driver age and gender

Although driver age was not associated with injury severity in the multivariable model (χ^2^ = 0.9, *p* = 0.48) (Table 1), younger occupants were more likely to be transported by younger drivers, with 55.1 % of the young teen occupants travelling with a teen driver. The majority of drivers were male (59.8 %). Male drivers were more likely to be speeding than female drivers (5.9 % vs. 3.3 %, χ^2^=15.6, *p*=0.03). The majority of drivers (67.9 %) involved in a side crash with a rear-seated occupant were under age 45 (Table 1).

#### Driver restraint use

Driver restraint use was strongly predictive of passenger restraint use, with passengers of a belted driver being more likely to be belted compared to passengers of drivers who were not belted (93.7 % vs. 28.3 %, χ^2^=517.9, *p*<0.0001) (not shown). Passengers of an unbelted driver were approximately 2 times more likely to be severely/fatally injured compared to passengers of a belted driver (Table 2). The majority of drivers (71.8 %) who were positive for alcohol involvement were categorized based on police reports and did not have an actual blood alcohol concentration reported. Driver alcohol involvement was associated with lower restraint use of passengers compared to passengers of drivers with no alcohol (77.1 % vs. 92.6 %, χ^2^= 24.7, *p*=0.0001) (not shown). Passengers transported by a driver positive for alcohol were more likely to have a severe/fatal injury compared to drivers negative for alcohol (OR: 11.98, 95 % CI: 9.57–14.99) (Table 2).

### Vehicle characteristics

#### Vehicle model year

The majority of vehicles (91.6 %) were model year 1998 or later (Table 1). Model year categorized by year of major vehicle safety improvements, was not significantly associated with injury severity in rear-seated occupants in either the unadjusted or belt-status adjusted model (Table 2).

#### Vehicle type

The distribution of occupants in vehicles was 57.8 % in cars, 19.7 % in SUVs, 10.4 % in vans, and 12.1 % in pick-up trucks (Table 1). Larger vehicles, such as SUVs, vans and pick-up trucks were protective compared to smaller vehicles in both unadjusted and adjusted models (Table 2).

### Crash characteristics

#### Crash type

The majority of side crashes were angle crashes (45.6 %), followed by sideswipe in the same direction (36.1 %) and sideswipe in the opposite direction (4.6 %) (Table 1). Occupants of vehicles involved angle crashes were nearly 11 times more likely to be seriously/fatally injured compared to opposite direction sideswipe crashes (OR: 10.85, 95 % CI: 9.24–12.73) (Table 2). Half of side crashes occurred on the same side as the occupant was seated (Table 1). Compared to occupants seated on the opposite side of the crash, occupants of same side were more likely to be severely/fatally injured after adjusting for restraint use (OR: 2.54, 95 % CI: 2.31–2.79) (Table 2).

#### Vehicle and struck type

The majority (82.7 %) of side crashes were two-vehicle crashes, with 57.1 % of crashes involving a similar-sized or smaller vehicle, while 25.7 % of vehicles involved in a side crash were struck by a larger vehicle (Table 1). Single vehicle crash, being struck by a larger vehicle and multiple vehicle involved crashes were associated with higher serious/fatal injury in both unadjusted and belt status adjusted models (Table 2).

#### Excessive speed

Excessive speed was associated with increased odds of severe or fatal injury in rear-seated occupants (OR: 5.70, 95 % CI: 4.66–6.97) (Table 2).

#### Rollover and ejection

Rollover occurred in 2.4 % of side crashes (Table 1). Half (50.3 %) of the rollovers were single vehicle crashes. Among those rollovers that involved two vehicles, 67.8 % were angle collisions. Occupants involved in a rollover were less likely to be belted compared to occupants not involved in a rollover (72.8 % vs. 92.7 %, χ^2^= 60.3, *p*<0.0001) (not shown). Among vehicles with a rollover, unbelted occupants were more likely be ejected (28.6 % vs. 0.2 %, χ^2^= 48.8, *p*<0.0001) and to have a serious/fatal injury (24.3 % vs. 5.5 %, χ^2^= 17.2, *p*<0.0001) (not shown). After adjusting for seat-belt use, occupants involved in a rollover continued to be more likely to be severely/fatally injured compared to occupants who were not involved in a rollover (OR: 23.17, 95 % CI: 5.22–102.85) (Table 2). Independent predictors of injury in rollovers and non-rollovers are reported separately in Table 2.

### Subpopulation analysis of same side crashes with and without vehicle safety ratings

**Fig. 3:**
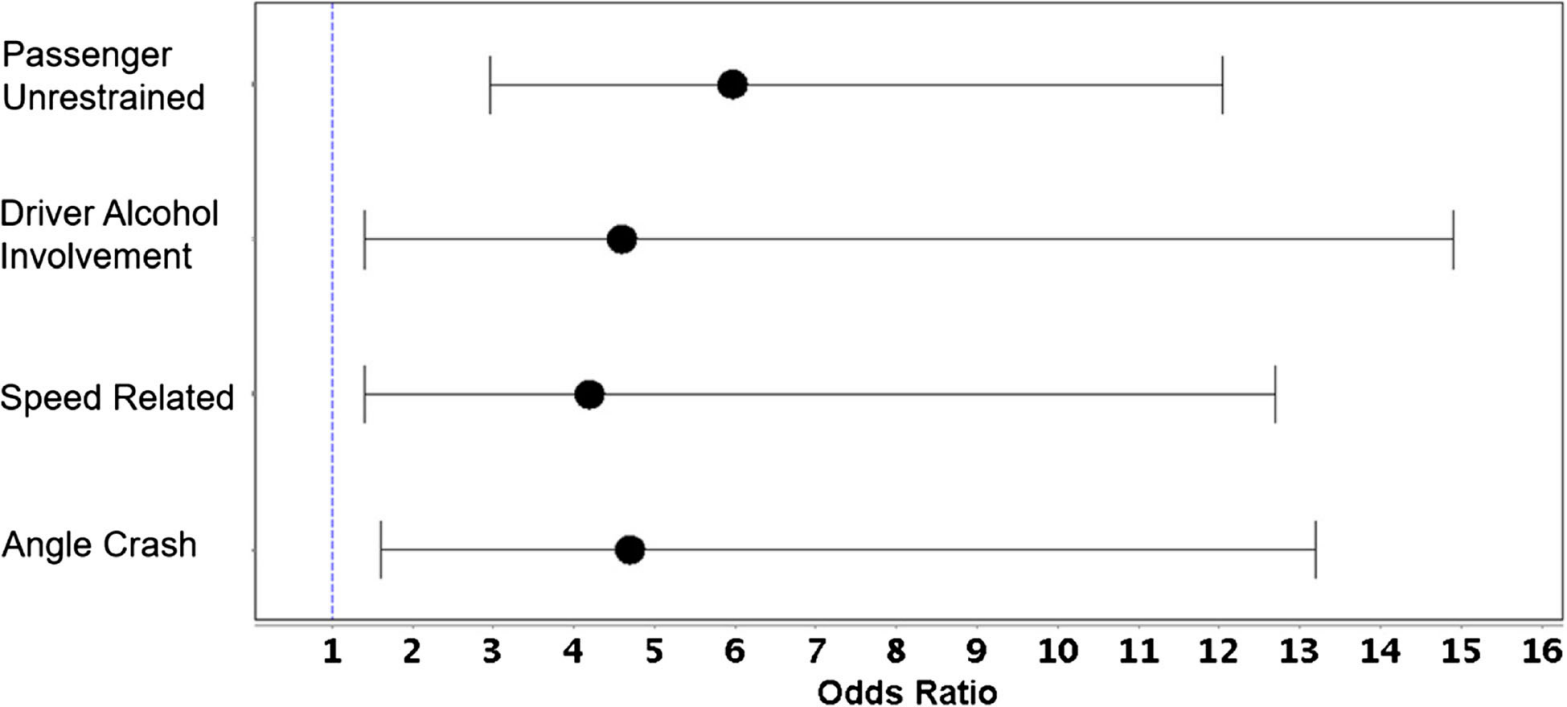
Subpopulation analysis of rear-seated adult occupants involved in same side crashes: adjusted odds ratios (with 95 % CIs) using multivariable logistic regression for serious/fatal injury of occupants, NASS/GES, 2011–2014. Adjusted for passenger age, passenger gender, and driver restraint status

A subpopulation of rear-seated occupants involved in a same-side crash was examined (*n*=1,971, weighted: 184,137). Passenger belt status, driver belt status, driver alcohol involvement, angle crash type and excessive speed were associated with increased injury severity. Unrestrained occupants were 5.97 times more likely to be severely/fatally injured compared to restrained occupants. Similarly, driver alcohol involvement was associated with increased rear-seated occupant injury severity, (OR: 4.61, 95 % CI: 1.42–14.98) as was speeding (OR: 4.17, 95 % CI: 1.37–12.69) (Fig. 3).

Approximately one-fifth (21.5 %) of vehicles involved in a same-side crash had a side crash safety rating (*n*=411, weighted: 39,208). Among those with a side crash safety rating, 52.6 % were rated good, 8.7 % were rated acceptable, 9.3 % were rated marginal and 29.8 % were rated poor (Table 3). The proportion of rear-seated occupants seriously/fatally injured by vehicle safety rating was 1.9 % for those travelling in vehicle rated as good, 8.2 % for acceptable, 3.2 % for marginal, 1.6 % for poor and 1.9 % for unrated (Table 3).

**Table 3:** Side crash ratings for passengers involved in same-side crashes, stratified by rear-seated injury severity for vehicle models from 1997–2014

	No/minor injury	Severe/fatal Injury	Total	
	Weighted (%)	Weighted (%)	Weighted	Chi-square
Rating				7.5 (0.062)
Unrated	139,126 (78.5)	2,682 (73.2)	141,809 (78.3)	
Poor	11,496 (6.5)	188 (5.1)	11,684 (6.5)	
Marginal	3,372 (1.9)	114 (3.1)	3,486 (1.9)	
Acceptable	3,137 (1.8)	282 (7.7)	3,419 (1.9)	
Good	20,221 (11.4)	398 (10.9)	20,619 (11.4)	

## Discussion

Initial point of impact and crash side relative to occupant seating position plays an important role in injury severity for rear-seated occupants involved in a side crash. Angle crashes and sideswipe in the opposite direction were associated with higher injury rates than sideswipe in the same direction. Occupants seated on the same side of the crash were more likely to have severe/fatal injury compared to occupants seated on the opposite side or in the middle seat after controlling for seat belt status. Although not specific to rear-seated occupants, this is consistent with previous studies that report a higher probability of injury in occupants seated on the near-side compared to occupants seated on the far-side (Newgard et al. 2005; Ryb et al. 2009). Raneses and Pressley (2015) have reported similar findings of rear-seated adult passengers who were same-side seated having higher mortality using FARS data.

Occupants of larger vehicles were less likely to be injured in a side-crash. This is consistent with previous studies conducted on front-seated passengers and drivers (Anderson 2008; Gayer 2004; Farmer et al. 1997). Larger vehicles have been reported to be more protective of front-seated drivers in collisions in side crashes (Gabler 2003; Vander Lugt D 1999).

Seat belt use was highly protective for serious/fatal injury for side crashes after controlling for seating position and crash impact type for side crashes. This is consistent with previous studies that report seat belt use being highly protective (Mayrose and Priya 2008; Zhu et al. 2007). A previous study of fatal collisions using FARS data demonstrated an exponential increase in mortality over the adult age span and an increased use of seatbelts in older occupants who were also more likely to die (Raneses and Pressley 2015). However, our study that included both fatal and nonfatal crashes and a younger population contains a much smaller proportion of fatal injuries and indicates that seatbelt use is highly protective for rear-seated occupants involved in same-side crashes. This may be due to the fact that our study population included both fatal and non-fatal crashes and our population was much younger compared to the previous study. In our study, mortality was examined in a category with serious injury specifically due to the small proportion of deaths captured in the NASS/GES dataset.

Previous studies report that newer model vehicles are protective for front-seated occupants (Ryb et al. 2011; Wenzel 2013; Glassbrenner 2012). In our study, model year was associated with less severe injury, with newer cars generally being safer compared to older cars only when analyzed as continuous variable. However, when analyzed categorically (Ryb et al. 2011), newer vehicles were not significantly associated with reduced injury severity in rear-seated occupants. This finding is likely due to improvements in vehicle engineering that focused on the driver with fewer safety improvements specifically geared to rear-seated occupants (Durbin et al. 2015, Mitchell et al. 2015).

Previous studies note that vehicle side crash ratings are protective for front-seated occupants (Teoh and Lund 2011). In our study of rear-seated occupants, side crash ratings were available for a small number, about one-fifth, of vehicles involved in a same-side crash and were not found to be associated with injury severity.

This study has limitations. Firstly, NASS/GES injury data uses officer reported KABCO and may not accurately reflect moderate and minor injuries. There are reports that use of KABCO injury rates may result in misclassification for degrees of severity of injury (Farmer 2003). To address this issue, we collapsed injury categories into serious/fatal injury vs. no or minor/possible injury instead of examining each injury severity level separately. Secondly, other studies reported that the effect of belt usage on severe/fatal injury can be overestimated, due to nonfatal crash victims being more likely to report restraint use inaccurately to law enforcement (Clark 2003; Robertson 1992). Because estimation of mortality using NASS/GES needs to be interpreted with caution (Clark 2003), we combined serious/fatal injury into one category which may be less biased. It is possible that the NASS/GES sampling scheme may underestimate fatality in older populations when compared to FARS. In this study, we believe that the finding of categorical age in older populations showing less severe/fatal injury in the older occupants compared to the middle - aged occupants is a dataset anomaly. In contrast, when examined continuously, older age was associated with increased severe/fatal injury. Thirdly, the majority of vehicles, especially older vehicles were not rated for side crash safety. In the subpopulation analysis of vehicles with safety ratings, we did not control for other factors that might be potential confounders for safety ratings and injury severity for rear-seated occupants due to a small sample size. Furthermore, driver alcohol involvement was categorized mainly based on law enforcement officer report instead of actual blood alcohol level as the majority of drivers did not have a blood alcohol concentration reported in the dataset. Lastly, we did not have information on side air bag deployment, which may have an impact on injury severity in side crashes.

## Conclusion

In summary, our results indicate that the initial point of impact relative to occupant seating position and crash type play important roles in rear-seated occupant safety. The results of our study also indicate that seat belt use is highly protective for all rear-seated adult occupants involved in a side-crash, including those involved in a same-side crash. Further study is needed to better understand the association between vehicle safety ratings and injury severity for rear-seated occupants.

## References

[CR1] Anderson M (2008). Safety for whom? The effects of light trucks on traffic fatalities. J Health Econ.

[CR2] Clark DE (2003). Effect of population density on mortality after motor vehicle collisions. Accid Anal Prev.

[CR3] Durbin DR, Jermakian JS, Kallan MJ, McCartt AT, Arbogast KB, Zonfrillo MR, Myers RK (2015). Rear seat safety: variation in protection by occupant, crash and vehicle characteristics. Accid Anal Prev.

[CR4] Evans L, Frick MC (1988). Seating position in cars and fatality risk. Am J Public Health.

[CR5] Farmer CM (2003). Reliability of police-reported information for determining crash and injury severity.

[CR6] Farmer CM, Braver ER, Mitter EL (1997). Two-vehicle side impact crashes: the relationship of vehicle and crash characteristics to injury severity. Accid Anal Prev.

[CR7] Gabler HC (2003). The evolution of side crash compatibility between cars, light trucks and vans. Evolution.

[CR8] Gayer T (2004). The fatality risks of sport-utility vehicles, vans, and pickups relative to cars. J Risk Uncertain.

[CR9] Glassbrenner D (2012). An analysis of recent improvements to vehicle safety.

[CR10] IIHS (2014). Ratings.

[CR11] IIHS (2014). Side impact crashworthiness evaluation guidelines for rating injury measures (version III).

[CR12] Mayrose J, Priya A (2008). The safest seat: effect of seating position on occupant mortality. J Saf Res.

[CR13] Mitchell RJ, Bambach MR, Toson B (2015). Injury risk for matched front and rear seat car passengers by injury severity and crash type: An exploratory study. Accid Anal Prev.

[CR14] National Safety Council (1990). Manual on Classification of Motor Vehicle Traffic Accidents.

[CR15] Newgard CD, Lewis RJ, Kraus JF, McConnell KJ (2005). Seat position and the risk of serious thoracoabdominal injury in lateral motor vehicle crashes. Accid Anal Prev.

[CR16] NHTSA (2015). 2014 FARS/NASS GES Coding and Validation Manual.

[CR17] Raneses E, Pressley JC (2015). Factors associated with mortality in rear-seated adult occupants involved in fatal motor vehicle crashes on US roadways. Injury epidemiology.

[CR18] Robertson LS (1992). The validity of self-reported behavioral risk factors: seatbelt and alcohol use. J Trauma.

[CR19] Ryb GE, Dischinger PC, Braver ER, Burch CA, Ho SM, Kufera JA (2009). Expected differences and unexpected commonalities in mortality, injury severity, and injury patterns between near versus far occupants of side impact crashes. J Trauma Acute Care Surg.

[CR20] Ryb GE, Dischinger PC, McGwin G, Griffin RL (2011). Crash-related mortality and model year: are newer vehicles safer? Annals of Advances in Automotive Medicine/Annual Scientific Conference.

[CR21] Samaha RR, Elliott DS (2003). NHTSA side impact research: motivation for upgraded test procedures. Eighteenth International Technical Conference on the Enhanced Safety of Vehicles, Paper.

[CR22] SAS Institute Inc (2014). Base SAS® 9.4 Procedures Guide.

[CR23] Smith KM, Cummings P (2006). Passenger seating position and the risk of passenger death in traffic crashes: a matched cohort study. Injury Prevention.

[CR24] Stewart TC, McClafferty K, Shkrum M, Comeau JL, Gilliland J, Fraser DD (2013). A comparison of injuries, crashes, and outcomes for pediatric rear occupants in traffic motor vehicle collisions. J Trauma Acute Care Surg.

[CR25] Sunnevang C, Rosen E, Bostrom O (2009). Real-life fatal outcome in car-to-car near-side impacts—implications for improved protection considering age and crash severity. Traffic injury prevention.

[CR26] Teoh ER, Lund AK (2011). IIHS side crash test ratings and occupant death risk in real-world crashes. Traffic injury prevention.

[CR27] Vander-Lugt D, Connolly T, Bhalsod D. Vehicle compatibility—analysis of the factors influencing side impact occupant injury. In: Proceedings of the SAE International Congress and Exposition. Detroit, MI, 1999.

[CR28] Wenzel T (2013). The effect of recent trends in vehicle design on US societal fatality risk per vehicle mile traveled, and their projected future relationship with vehicle mass. Accid Anal Prev.

[CR29] Zhu M, Cummings P, Chu H, Cook LJ (2007). Association of rear seat safety belt use with death in a traffic crash: a matched cohort study. Injury Prevention.

